# NADPH homeostasis in cancer: functions, mechanisms and therapeutic implications

**DOI:** 10.1038/s41392-020-00326-0

**Published:** 2020-10-07

**Authors:** Huai-Qiang Ju, Jin-Fei Lin, Tian Tian, Dan Xie, Rui-Hua Xu

**Affiliations:** 1 State Key Laboratory of Oncology in South China, Collaborative Innovation Center for Cancer Medicine, Sun Yat-sen University Cancer Center, Sun Yat-sen University, 510060 Guangzhou, China; 2 Research Unit of Precision Diagnosis and Treatment for Gastrointestinal Cancer, Chinese Academy of Medical Sciences, 510060 Guangzhou, China

**Keywords:** Cancer metabolism, Drug development

## Abstract

Nicotinamide adenine dinucleotide phosphate (NADPH) is an essential electron donor in all organisms, and provides the reducing power for anabolic reactions and redox balance. NADPH homeostasis is regulated by varied signaling pathways and several metabolic enzymes that undergo adaptive alteration in cancer cells. The metabolic reprogramming of NADPH renders cancer cells both highly dependent on this metabolic network for antioxidant capacity and more susceptible to oxidative stress. Modulating the unique NADPH homeostasis of cancer cells might be an effective strategy to eliminate these cells. In this review, we summarize the current existing literatures on NADPH homeostasis, including its biological functions, regulatory mechanisms and the corresponding therapeutic interventions in human cancers, providing insights into therapeutic implications of targeting NADPH metabolism and the associated mechanism for cancer therapy.

## Background

In cancer cells, the appropriate levels of intracellular reactive oxygen species (ROS) are essential for signal transduction and cellular processes.^1,2^ However, the overproduction of ROS can induce cytotoxicity and lead to DNA damage and cell apoptosis.^3^ To prevent excessive oxidative stress and maintain favorable redox homeostasis, tumor cells have evolved a complex antioxidant defense system that strategically adjusts multiple antioxidant enzymes such as catalase, glutathione reductase, and antioxidant molecules. The latter are dependent on the generation of nicotinamide adenine dinucleotide phosphate (NADPH), which is used to maintain reduced glutathione (GSH) and thioredoxin (TRX).^4–6^ NADPH is also well known as an essential electron donor and an indispensable cofactor that is used for transferring and reserving reduction potential for numerous anabolic reactions.^7^

NADP(H) is predominantly bound to intracellular proteins with different affinities.^8^ The intracellular content of NADP(H) differs markedly among tissues and cell types. For instance, the total NADP(H) is about 420 nmol/g wet weight in rat liver and 59% of total NADP(H) is found in mitochondria, and 30 nmol/g wet weight in skeletal muscle,^5,8^ and the NADPH concentration in the cytosol is 3.1 ± 0.3 and 37 ± 2 µM in the mitochondrial matrix in HeLa cells.^9^ In addition, the redox potentials of the mitochondrial and cytosolic NADP(H) systems are the same around—400 mV in the liver.^8^

A growing body of evidence has shown that regeneration and maintenance of the cellular NADP(H) content is strongly implicated in a variety of pathological conditions, such as diabetes, cardiovascular disease, neurodegenerative diseases, aging,^4,5^ especially in tumorigenesis and cancer progression.^10^ Compared with non-tumor cells, tumor cells usually maintain high levels of NADPH, not only to power redox defense but also to use for biosynthetic reactions to sustain their rapid growth.^5,11^ This realization has prompted molecular studies of NADPH metabolism and its exploitation for the development of anticancer agents. Recent advances have revealed that therapeutic modulation based on NADPH metabolism has been widely viewed as a novel and effective anticancer strategy.

In this review, we summarize the current existing literatures on NADPH metabolism, including its biological functions, regulatory mechanisms, and the corresponding therapeutic interventions directly or indirectly targeting NADPH metabolism in cancer.

## NADPH-dependent biological functions in cancer

**Fig. 1:**
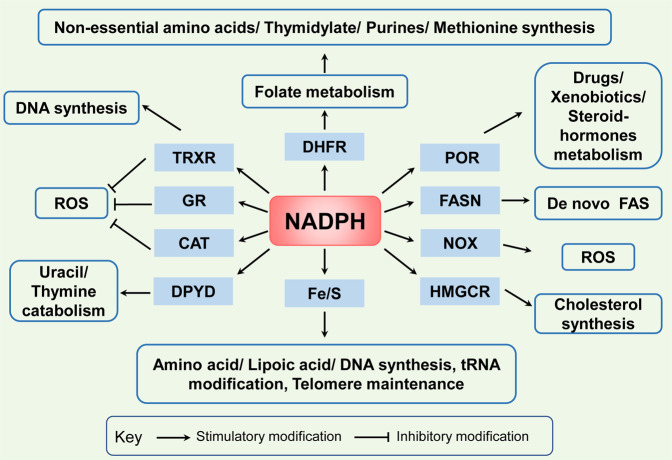
NADPH-dependent biological functions in cancer. There are three principal ways in which NADPH is used. First, NADPH is an essential cofactor of glutathione reductase (GR) and TRXR in GSH and TRX-peroxiredoxin (PRX) system, respectively, and reactivates catalase (CAT) to deactivate ROS for antioxidation; Second, NADPH is a crucial electron source for DHFR, Fe/S, POR, FANS, HMGCR, DPYD contributing to several reductive synthesis reactions, such as FAS, non-essential amino acids, nucleotides, and steroids synthesis; Third, NADPH is a substrate for NOXs to generate ROS

Both NAD(H) and NADP(H) are cofactors that are used for transferring and reserving reduction potential.^7,9^ Although the structures are closely related, NAD(H) and NADP(H) are recognized by unique compartmentalized enzymes and exert different functions. NAD(H) is mainly involved in catabolic reactions,^5,12,13^ whereas NADP(H) is primarily involved in cellular antioxidative effects and anabolic reactions as shown in Fig. 1.

### Antioxidative effects

In cancer cells, overcoming oxidative stress is a critical step for tumor progression. NADPH plays a key role in cellular antioxidation systems by providing reducing equivalents to generate reduced forms of antioxidant molecules, which are highly corrected with cancer cell biological behaviors.^14^ On the one hand, GSH reductase converts GSSG to GSH using NADPH as an important cofactor, then GSH acts as a cosubstrate for GSH peroxidase (GPX) that reduces hydrogen peroxide (H_2_O_2_) and other peroxides to H_2_O or alcohol to deactivate ROS.^15,16^ On the other hand, TRX reductase (TRXR) utilizes NADPH as an electron donor to maintain the reduced form of TRX, which contributes to scavenge H_2_O_2_ and reduce ribonucleotide reductase (RNR) for DNA synthesis.^17,18^ In addition, in some cell types, NADPH binds to the important H_2_O_2_-disposing enzyme: catalase, and reactivates it when it has been inactivated by H_2_O_2_.^19^

### Reductive synthesis

NADPH is also a crucial electron source for several reductive synthesis reactions, including fatty acids, amino acids, nucleotides, and steroids synthesis to sustain rapid tumor cell growth.^20^ Primarily, NADPH provides reducing equivalents for fatty acid synthase (FASN), the main rate-limiting enzyme, to synthesize fatty acids with acetyl-CoA serving as a primer and malonyl-CoA as a two-carbon donor,^21,22^ and provides the needed electrons for iron–sulfur (Fe/S) protein assembly that participate in non-essential amino acid biosynthesis and lipoic acid synthesis, tRNA modification, DNA replication and repair, and telomere maintenance.^23^ NADPH is also needed for dihydrofolate reductase (DHFR) enzyme to catalyze the reduction of dihydrofolate to tetrahydrofolate (THF) in folate metabolism, which is required for de novo biosynthesis of thymidylate, purines, methionine, and some amino acids.^24^ Besides, NADPH acts as the reducing reagent for 3-hydroxy-3-methylglutaryl-coenzyme A reductase (HMGCR), the rate-limiting enzyme of the mevalonate pathway, which leads to the synthesis of cholesterol and nonsterol isoprenoids.^25^ NADPH also acts as a cosubstrate for dihydropyrimidine dehydrogenase (DPYD), which catalyzes the reduction of uracil and thymine to 5,6-dihydrouracil and 5,6-dihydrothymine, respectively.^26^ In addition, the activity of the cytochrome P450 reductase (POR) also requires NADPH, which has a major role in the metabolism of drugs, xenobiotics, and steroid hormones.^27^

### Free radical generation

In addition, NADPH is also responsible for the generation of free radicals by NADPH oxidases (NOX) as a substrate. NOXs (NOX1–5 and dual oxidases (DUOX) 1 and 2) catalyze the superoxide anions or H_2_O_2_ from NADPH and oxygen.^28–30^ NOX-mediated ROS broadly and specifically regulate various redox-sensitive signaling pathways involved in cancer progression via stimulating oncogenes, such as Src and Ras, and inactivating tumor suppressor proteins, such as TP53 and PTEN.^31^

## Molecular mechanisms of NADPH homeostasis in cancer

**Fig. 2:**
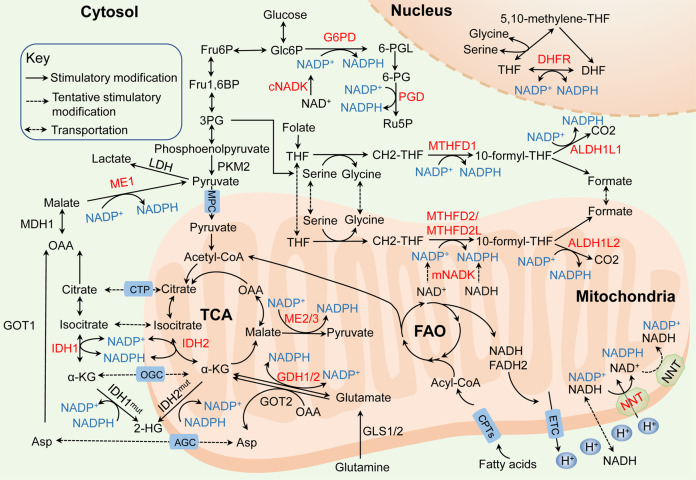
Molecular mechanisms of NADPH homeostasis in cancer. The principal generation of NADPH (blue) with dysregulated pathways and enzymes (red) in cancer: (i) NADKs catalyze the phosphorylation of NAD(H) to form NADP(H) via the de novo synthesis (cNADK in the cytosol and mNADK in mitochondria). (ii) the pentose phosphate pathway (PPP) utilizes G6PD and PGD to maintain the cytosolic NADPH. (iii) the folate-mediated one-carbon metabolism reduces NADP^+^ to NADPH by MTHFD1/ALDH1L1 in the cytosol, MTHFD2/MTHFD2L/ALDH1L2 in mitochondria and DHFR in the nucleus. (iv) IDH1 located in the cytosol and IDH2 located in mitochondria generate NADPH, but mutant IDHs consume NADPH. (v) ME1 located in the cytosol and ME2/3 located in mitochondria convert NADP^+^ into NADPH; (vi) the glutamine metabolism generates NADPH by GDH1/2 directly in mitochondria and generates aspartate that is transported into the cytosol for NADPH production depending on ME1. (vii) NNT catalyzes the transfer of hydride ions from NADH to NADP^+^ and produces NADPH to maintain the mitochondrial NADPH and the reverse-mode NNT that consumes NADPH may exist in cancer cells. (viii) The CPT1/2-mediated FAO generates acetyl CoA that enters the TCA cycle and contributes to NADPH production depending on IDHs and MEs. MPC mitochondirial pyruvate carrier, CTP citrate transport protein, OGC α-ketoglutarate-malate carrier, AGC aspartate–glutamate carrier

Understanding NADPH production and consumption routes is essential to a global understanding of cancer metabolism. As shown in Fig. 2, the NADPH homeostasis is mainly regulated by several metabolic pathways and enzymes including NAD kinase (NADK), the pentose phosphate pathway (PPP), the folate-mediated one-carbon metabolism, malic enzymes (ME), the nicotinamide nucleotide transhydrogenase (NNT), cytosolic or mitochondrial NADP-dependent isocitrate dehydrogenase (IDH1 and IDH2), the glutamine metabolism, and the fatty acid oxidation (FAO). However, for the general NADPH generation in cells, the relative contribution of these pathways and enzymes to NADPH production remains elusive. Recent study show that cellular NADPH could be largely generated by PPP, the folate-mediated one-carbon metabolism and ME in cancer and proliferation cells.^32,33^ Also, mounting evidence suggests that these different processes and enzymes have functional connections for NADPH homeostasis in cancer. For instance, FAO accelerates the TCA cycle to produce citrate, which is exported to the cytosol to engage in NADPH production through ME1 and IDH1.^34^ Here we review current knowledge of the underlying mechanisms of NADPH homeostasis following its de novo synthesis, relative contribution of related enzymes and pathways in cancer.

### NAD kinase

NADPH de novo synthesis is catalyzed by NADKs, which catalyze the phosphorylation of NAD^+^ to form NADP^+^. Subsequently, the dehydrogenases/reductases in various metabolic pathways convert NADP^+^ into NADPH.^10,12^ NADKs are found in almost all human organs except skeletal muscle, and localized in both cytosol and mitochondria. Compared to cytosolic NADK (cNADK), mitochondrial NADK (mNADK) has a distinctive feature that it can directly phosphorylate nicotinamide adenine dinucleotide (NADH) to generate NADPH to alleviate oxidative stress in mitochondria.^35^

The Cancer Genome Atlas (TCGA) database indicates both cNADK overexpression and the presence of several cNADK mutants in multiple tumor types.^10^ Notably, a novel cNADK mutant, NADK-I90F, is found in pancreatic ductal adenocarcinoma cancer (PDAC) patients. CNADK-I90F has a lower *K*_m_ and higher *V*_max_ for NAD^+^ compared to wild-type cNADK, which indicates increased enzyme activity. Consistently, compared with cNADK wild-type cells, cells expressing cNADK-I90F have elevated NADPH levels and reduced ROS levels.^36,37^ In addition, in diffuse large B-cell lymphoma (DLBCL) and colon cancer, silencing cNADK with shRNA impairs the pool of NADPH and suppresses cancer cell growth.^38^ In terms of NADKs activities, cNADK phosphorylated at S44, S46, and S48, which may be mediated by the phosphoinositide 3-kinase (PI3K)–Akt signaling, has enhanced activity in breast cancer and lung cancer cells, thereby increasing NADPH production.^39^ Based on its recent discovery, the relevant role of mNADK in human cancers still need to be clarified, but the wild-type and mutant cNADK are potential clinical targets for cancer therapy.

### Pentose phosphate pathway

The PPP diverges at the first step of glycolysis, which serves as the largest contributor of cytosolic NADPH and NADPH generation undergoes three irreversible reactions in the PPP oxidative branch.^40–42^ Studies have proved that NADPH production is dramatically increased by enhancing the flux of glucose into the PPP oxidative branch in various cancers.^43,44^ Glucose-6-phosphate dehydrogenase (G6PD) that exists as either an active dimer or an inactive monomer dehydrogenates G6P to yield 6-phosphogluconolactone (6-PGL) and NADPH in the first reaction. Then, 6-phosphogluconate dehydrogenase (PGD) that often functions as a homodimer catalyzes the oxidative decarboxylation of 6-phosphogluconate (6-PG) to synthesize ribulose-5-phosphate (Ru5P) and a second NADPH in the third reaction.^45,46^

Increasingly, more studies have shown that G6PD activity is increased in several types of cancers, including bladder, breast, prostate, gastric cancers compared with normal tissues, and the high expression of G6PD predicts poor clinical outcome in various cancer patients and plays critical roles in tumorigenesis and chemoresistance.^47,48^ PGD is also hyperactive and plays a fundamental role in tumor growth.^49,50^ G6PD or PGD depletion significantly decrease NADPH levels and enhance chemotherapeutic drugs-induced cell apoptosis by redox modulation.^51,52^ For what concerns activity regulation, NADP^+^ is required for G6PD enzymatic activity, whereas NADPH negatively regulates its activity. Hence, tumor cells with higher NADPH consumption exhibit higher levels of active G6PD.^45^ Interestingly, a study also shows that NADPH level is not changed by silencing PGD expression, which is possible that a temporally increased NADP^+^/NADPH ratio compensatory increased G6PD activity, thus generating NADPH.^45^

The NADPH homeostasis is also regulated by the rate-limiting enzyme activity affected by the posttranslational modification. Studies indicate that the glycosylation, SIRT5-mediated deglutarylation and SIRT2-mediated deacetylation all enhance G6PD activity and maintain cellular NADPH homeostasis.^53–55^ Both the phosphorylation of PGD at Y481 upon EGFR activation and acetylation of PGD at K76 and K294 by acetyltransferases enhance its activation for producing NADPH in cancer cells.^56,57^ Conversely, protein kinase A (PKA) inhibits G6PD activity by directly phosphorylating it on serine and threonine residues.^58^ Additionally, G6PD activity can be regulated by several signaling pathways in tumors, such as the PI3K/AKT, Ras, Src, Nrf2, mTORC1, PETEN, ATM, and TP53 pathways, in a direct or indirect manner (reviewed in refs. ^45,47^). For instance, the PTEN protein and cytosolic TP53 bind to G6PD to prevent the assembly of G6PD monomers into active dimers and thus decease the PPP flux.^59,60^

### Folate-mediated one-carbon metabolism

Folate-mediated one-carbon metabolism has been long recognized and attributed to its function of producing one-carbon units for nucleic acid and methionine synthesis, another crucial function of this pathway is generating reducing power NADPH.^61,62^ Serine and glycine are the major carbon sources of this pathway. The activation of serine biosynthesis pathway enhances NADPH generation in cancer cells.^63^ Conversely, eliminating serine from the medium decreases the NADPH/NADP^+^ ratio and impairs cancer cell growth.^64^ Methylene tetrahydrofolate dehydrogenases (MTHFD1 in cytosol and MTHFD2 or MTHFD2L in mitochondria) catalyze the oxidation of 5,10-methylene-THF (CH2-THF) to form 10-formyl-THF, and 10-formyl-THF dehydrogenases (ALDH1L1 in cytosol and ALDH1L2 in mitochondria) catalyze the oxidization of 10-formyl-THF to generate CO_2_ with concomitant NADPH production. In the nucleus, the THF carrier is oxidized to DHF in an NADPH-generating reaction with electrons used to reduce one-carbon units to the methyl level.^65–67^

MTHFD2 is postulated to be the “main switch” that produces additional one-carbon units in mitochondria to enable rapid growth.^63^ The expression of MTHFD2 is closely related to the response of the folate antagonist methotrexate (MTX) and the thymidylate synthase inhibitor pemetrexed.^68,69^ Both MTHFD2 and MTHFD1 are markedly elevated and correlated with poor survival across human cancers.^70–72^ Moreover, study indicates that combining serum AFP with MTHFD1 enhances the prognostic prediction accuracy in hepatocellular carcinoma (HCC).^73^ Quantitative flux analysis reveals depletion of either MTHFD2 or MTHFD1 results in decreased cellular NADPH/NADP^+^ and GSH/GSSG ratios and increased cell sensitivity to oxidative stress.^32^ Suppression of MTHFD2 disturbs redox homeostasis, accelerates cell death in both colorectal cancer (CRC),^74,75^ and acute myeloid leukemia (AML).^64^ MTHFD2 is also critical for cancer stem-like properties and chemoresistance, suggesting that disturbing NAPDH homeostasis may prevent recurrence and eradicate tumors.^76^ And, MTHFD1 depletion reduces both the frequencies of circulating melanoma cells in the blood and metastatic disease burden in mice bearing melanoma,^77^ suggesting that NAPDH homeostasis represents therapeutic targets to impede distant metastasis. However, the association between MTHFD2L, which can use either NAD^+^ or NADP^+^ for dehydrogenase activity, and tumors remains to be investigated.

Cytosolic ALDH1L1 mainly regulates reduced folate pools and purine biosynthesis, while mitochondrial ALDH1L2 produces NADPH in response to oxidative stress.^78^ Although ALDH1L1 is overexpressed in NSCLC and GC cancer,^79,80^ ALDH1L1 is reported profoundly downregulated or silenced in cancers, rendering it a candidate tumor suppressor.^81,82^ Nevertheless, ALDH1L2 is highly expressed and presents as an independent prognostic factor for overall survival in melanoma, PDAC, and CRC.^77,78,83^ Depletion of ALDH1L2 markedly decreases the NADPH/NADP^+^ and GSH/GSSG ratios, reduces the circulating tumor cells in blood and alleviates the metastatic burden.^77,83,84^ In addition, the expression of ALDH1L2 is upregulated by some certain drugs, such as thapsigargin and tunicamycin, endoplasmic reticulum stress inducers in immortalized human B cells,^85^ mitotane, an adjuvant monotherapy used for treating adrenocortical carcinoma,^86^ and the indomethacin, an anti-inflammatory agent in breast cancer cells.^87^ Thus, further exploration of the association between the effects of these drugs on the ALDH1L2 expression and the cellular response to redox stress is needed.

### Malic enzymes

ME participate in reactions that link the components of catabolic metabolism in glycolysis and the Krebs cycle via the oxidative decarboxylation of malate to pyruvate, thereby inducing the anabolic metabolism with concomitant NADPH production.^32,88^ A quantitative flux analysis showed that the direct contribution of ME to NADPH generation was estimated to equal the contribution of the PPP.^89^ ME family consists of three isoforms: ME1 is located in the cytosol and ME2, ME3 are located in mitochondria. ME1 and ME3 require NADP^+^ and ME2 utilizes either NAD^+^ or NADP^+^ for their catalytic activities, thus NADPH can be produced by ME both directly and indirectly through the activity of the NNT that catalyzes the transfer of hydride ions from NADH to NADP^+^ and produces NADPH in mitochondria.^90^ However, ME1 and ME2 seem to be the main isoforms because ME3 is hardly negligibly detected in many assessed mammalian cells.^91^

The overexpression of ME1 is significantly associated with a poor prognosis for people with cancer, including those with gastric cancer, oral squamous cell carcinoma, breast cancer, lung cancer, etc.^92–95^ Silencing ME1 markedly reduces NADPH and increases ROS levels, ultimately induces cell apoptosis under oxidative stress, such as glucose starvation or anoikis.^96,97^ Moreover, the ME1 protein is hypophosphorylated at S336 and hyperacetylated at K337 by PGAM family member 5 and acetyl-CoA acetyltransferase, respectively, resulting in ME1 translocation from mitochondria to the cytosol, dimerization and activation, thus strongly promoting NADPH generation and tumorigenesis.^98^ ME1 expression is also regulated by well-known tumor suppressors or oncogenes such as TP53 or KRAS.^91,99^ Intriguingly, there is a direct crosstalk between ME1 and PPP components, and ME1 increases the ability of PGD to bind to 6-PG, enhancing NADPH generation.^100^

ME2 is also overexpressed in several cancers according to recent investigations, and is closely associated with cancer growth, metastasis, and poor outcomes.^101,102^ ME2 depletion, accompanied by an increased NADP^+^/NADPH ratio and ROS levels, impacts PI3K/AKT signaling and enhances the sensitivity of erythroleukemia and NSCLC cells to cisplatin.^103,104^ Besides, ME2 ablation results in elevated cellular ROS levels, which activates the AMPK pathway and then stimulates TP53 to attenuate melanoma cell proliferation.^105,106^ ME2 is frequently hemizygously codeleted along with tumor suppressor SMAD4 in human solid tumors including gastric cancer and PDAC.^107,108^ In ME2-unexpressed gastric cancer cells, its isoenzyme ME1 is upregulated to replenish intracellular NADPH and promotes cell survival under glucose starvation and anoikis.^107^ ME3 is in lower enzymatic activity than do ME2 in mitochondria. However, in ME2 homozygously deleted PDAC cell lines, its isoenzyme ME3 plays the compensatory roles for intracellular NADPH homeostasis.^108,109^ These findings provide a prime ‘collateral lethality’ therapeutic strategy for the treatment of a substantial fraction of GC or PDAC patients.

### Nicotinamide nucleotide transhydrogenase

NNT is an integral mitochondrial inner membrane protein in eukaryotes that catalyzes the transfer of hydride ions from NADH to NADP^+^ and produces NADPH utilizing the proton motive force generated by the electron transport chain (ETC).^110^ The process is essential for maintaining the mitochondrial NADPH and NADH pools. NNT activity contributes to 45% of the total NADPH in mitochondrial pool, indicating a significant role of NNT for NADPH pool maintenance,^111^ and NADPH obtained by NNT is also used for the reductive carboxylation of α-KG to isocitrate mediated by IDH2.^112^ In contrast to this prevailing view, a fascinating work illustrates that the NNT reverses the direction upon NADPH consumption to support NADH and ATP productions under a pathological workload, at the cost of NADPH-linked antioxidative capacity. The models unexpectedly show that lacking a functional NNT presents with less oxidative damage to the heart compared to mice with active NNT.^113^ This finding provides potentially fresh insights into pathology and metabolic regulation, but more study about the NNT reversal process in cancer is urgently needed.

In cancer cells, NNT activity is stimulated by hyperpolarized mitochondria. Further, the NADH from increased glycolysis in the cytosol can be transferred to mitochondria to drive NADH-dependent NNT.^89^ Additionally, NNT is overexpressed in gastric cancer cell, which is associated with lower overall survival and disease-free survival. NNT knockdown shows limited ability to maintain NADPH levels and reduces tumorigenicity under oxidative stress conditions, such as that induced by anoikis, glucose deprivation in vitro, or impairs peritoneal dissemination and lung metastasis in vivo.^114^ Similar effects are observed in liver cancer,^115^ pheochromocytoma^116^ and NSCLC,^111^ and NNT is likely to be activated by NADPH consumption, such as in IDH-mutant cells.^117^ Additionally, considered as a key antioxidative enzyme, NNT is critical for inducing macrophage inflammatory responses^118^ and preventing ROS-induced cytotoxicity in T cells exposed to asbestos that can cause a reduction in antitumor immunity.^119^ To date, NNT appears to play a key role in tumorigenesis and modification of NNT may regulate immune effects of anti-tumor. Unfortunately, pharmacological inhibitors specific for NNT have not been reported and need to be developed.

### Isocitrate dehydrogenases (IDH)

IDH also facilitates the generation of NADPH from NADP^+^ by catalyzing the oxidative decarboxylation of isocitrate to α-ketoglutarate (α-KG) for TCA cycle.^120^ There are three subtypes of IDH: IDH1 is located within the cytosol and peroxisomes, and IDH2/3 are primarily found in mitochondria. IDH1/2 use NADP^+^ as a cofactor and conduct a reversible reaction, while IDH3 uses NAD^+^ as a cofactor and conducts irreversible conversion.^121,122^

Multiple lines of evidences have revealed that IDH1 is overexpressed in numerous cancers and is closely correlated with poor prognoses of patients with non-small cell lung carcinoma (NSCLC),^123^ PDAC,^124^ or one of several hematological malignancies.^125^ Notably, ELISA demonstrate that IDH1 level is also significantly elevated in the plasma of NSCLC patients, suggesting that it can be used as a potential plasma biomarker.^126^ The upregulation of IDH1 may represent a common metabolic adaptation for diminishing oxidative stress and supporting macromolecular synthesis, consequently promoting tumor growth and therapy resistance.^125^ Furthermore, IDH1 silencing results in decreased NADPH and α-KG levels, with the increased ROS levels, leading to cancer cell apoptosis in NSCLC.^123^ Besides, oxidative stress conditions also increase the innately high IDH1 expression, and IDH1 silencing significantly enhances cell sensitivity to cancer chemotherapy, radiotherapy, and photodynamic therapy by reducing NADPH.^124,127,128^ In addition, IDH1 is hyperacetylated in CRC cells and is significantly correlated with distant metastasis and poor survival. SIRT2-dependent IDH1 deacetylation at K224 impairs its enzymatic activity and represses its malignant behaviors in CRC.^129^ Specially, studies also found that IDH1 is significantly downregulated in clear cell renal cell carcinoma (ccRCC) compared with normal kidney cells, suggesting that IDH1 may function as a candidate tumor suppressor for ccRCC.^130,131^

Most studies indicate that IDH2 is also significantly upregulated in ESCC,^132^ ovarian cancer,^133^ lung cancer and other types of cancer,^134^ playing a pro-oncogenic role. Overexpression of IDH2 decreases ROS levels and increases cancer cell growth.^121^ IDH2 depletion decreases the expression of HIF1α and leads to the attenuation of tumor growth in lung cancer.^134^ However, because of heterogeneity among cancer cells, other studies have shown that IDH2 expression is decreased in metastatic HCC and gastric cancer tissues compared with paired normal tissues.^135,136^ The underlying mechanism is that these cells lacking IDH2 show enhanced invasive behavior due to the increase in matrix metalloproteases, which depend on the NF-κB pathway. In addition, NAD^+^ production by the NNT enhance SIRT3-mediated deacetylation and loss of NAD^+^-dependent deacetylase SIRT3 increases the acetylation of IDH2 at K413 and decreases its enzymatic activity by reducing dimerization, thus regulates mitochondrial redox status and promotes cell tumorigenesis in luminal B breast cancer,^137^ and B cell malignancies.^138^ SIRT5-mediated IDH2 desuccinylation also regulates cellular NADPH homeostasis and redox potential.^54^

The contribution of IDH to NADPH generation in cancer remains controversial. IDH1 and IDH2 also catalyze the reductive carboxylation and support tumor cells growth with defective mitochondria. Studies show that IDH1/2 syntheses isocitrate/citrate from α-KG with NADPH consumption, then the isocitrate/citrate import into the mitochondria and contribute to suppress mitochondrial ROS.^139,140^ In addition, recently, IDH1 and IDH2 gene mutations have been prevalent in several diverse malignancies, including glioma, AML, angioimmunoblastic lymphomas, chondrosarcoma, and melanomas.^141,142^ Recurrent somatic mutation of residues are mainly located at enzymatic active sites that bind to isocitrate, typically at R132 including R132H, R132L, R132S, R132C, and R132G in IDH1, and R140Q or R172K in IDH2.^143,144^ The mutated IDH1 and IDH2 proteins are endowed with a novel ability to catalyze the reduction of α-KG to generate a rare metabolite, 2-hydroxyglutarate (2-HG), while consuming NADPH.^145^ Further, the relevance of these mutations and their roles in carcinogenesis and possible therapeutic implications have been extensively reviewed elsewhere.^141,146,147^

### Glutamine metabolism

Glutamine metabolism is a major cellular carbon source for the TCA cycle, a nitrogen donor for nucleotide, amino acid, and lipid biosynthesis, it is also critical for maintaining NADPH levels.^148,149^ Proliferating cancer cells exhibit aerobic glycolysis, leading to a shift in glucose carbon away from the TCA cycle, which results in the increased use of glutamine to fuel anabolic processes to support rapid cell growth with increased NADPH and ammonia generation. Glutaminolysis is the mitochondrial pathway by which glutamine is first deaminated to glutamate by glutaminases (GLS1/2). Then, either NADPH-dependent glutamate dehydrogenases (GDH) or other transaminases, including glutamate oxaloacetate transaminase 2 (GOT2) and glutamate pyruvate transaminase 2 (GPT2), convert glutamate into a-KG to meet the need for corresponding amino acids.^89^

Conventionally, GDH (coded by the GLUD gene) is the more predominant enzymes vital for the reactions needed to replenish the TCA cycle and yield NADPH than GOT2 and GPT2, which consists of ubiquitously expressed GDH1 and GDH2 mainly existing in neuronal and testicular tissue and having lower activity than GDH1.^150^ GDH1 is highly expressed in most tumor samples and correlated with tumor progression stage, including breast cancer and lung cancer cells.^151,152^ GDH1 depletion results in imbalanced redox homeostasis and cell cytotoxicity and attenuates cancer cell proliferation, which as well as the results in erythroleukemia cells, while it negligibly affects normal cell proliferation.^151^ Additionally, enhanced GDH1 activity has also been reported to be a possible prognostic marker and an indicator of metastasis in patients with CRC or gastric cancer.^153,154^ Under conditions of insufficient glycolysis caused by glucose deprivation, 2-deoxyglucose treatment or Akt signaling inhibition, glutamine-addicted cells are more sensitive to GDH1 deficiency.^155^ Furthermore, GDH-derived NADPH is consumed to support the reductive carboxylation of α-KG by IDH2, and the compensatory increase in the expression of GDH1 or GDH2 promote the growth of IDH-mutant glioma cells.^156^ Besides, with the consumption of extracellular glutamine, GDH can also catalyze ammonia derived from glutaminolysis and α-KG to support the synthesis of glutamate and downstream metabolites by reductive amination in a NADPH consumption manner to meet the cancer cells growth.^148,157,158^

Specifically, some cancer cells, such as PDAC and CRC cells, depend on a noncanonical glutamine metabolism pathway in the cytosol under the regulation of oncogenic KRAS activation. Glutamine-derived aspartate induced by GOT2 is transported into the cytosol and converted by GOT1 to oxaloacetate, then converted by malate dehydrogenase (MDH1) into malate and subsequently oxidized into pyruvate by ME1 to create NADPH.^159,160^ GHD1 shRNA has no effect on PDAC cells growth, while knocking down GOT2 elevates ROS levels and leads to cell senescence.^161^ Further, cytosolic GOT1 inhibition decreases oxaloacetate levels and reduces the cellular NADPH/NADP^+^ and GSH/GSSG ratios.^159^ Consistent with these findings, the addition of exogenous malate protects cells from excessive ROS accumulation in MDH1-knockdown cells.^162^ Consequently, targeting the glutamine metabolism pathway, which is essential for cancer cells but dispensable for normal cells, may lead to novel therapeutic approaches to treat refractory tumors.

### Fatty acid oxidation

In addition, FAO pathway is also key for providing NADPH indirectly, which is indispensable in many cancers especially under metabolic stress. FAO generates NADH, FADH2, and acetyl coenzyme A (CoA) in each round,^163^ and NADH and FADH2 enter the ETC while the acetyl CoA enters the TCA cycle to produce citrate, which is exported to the cytosol to engage in NADPH production through ME1 and IDH1.^34^ FAO and FAS are both essential for tumor progression and support each other. Acetyl CoA and NADPH accumulated from FAO metabolism in the cytosol are needed to initiate FAS.^164^ The carnitine palmitoyl transferases (CPT), the rate-limiting enzymes in the FAO pathway, transport long-chain acyl-CoA from the cytosol to mitochondria.^165^ CPT-mediated FAO activation is reported to play key roles in maintaining NADPH homeostasis and promoting cell metastasis and chemoresistance in gastrointestinal cancer^166,167^ and melanoma.^168^ Recent studies also show that knocking down PPAR coactivator 1α (PGC1α), an important transcriptional coactivator regulating CPT1A and CPT1B, obviously decreases the ratio of NADPH/NADP^+^ and ATP levels, impairing radiation resistance in nasopharyngeal carcinoma (NPC) cells.^169^ What’s more, AMP-activated protein kinase (AMPK) also regulates the function of FAO in maintaining NADPH homeostasis and promotes tumor cell survival under oxidative stress or metabolic stress.^170–173^

## Therapeutic implications for targeting NADPH metabolism

**Fig. 3:**
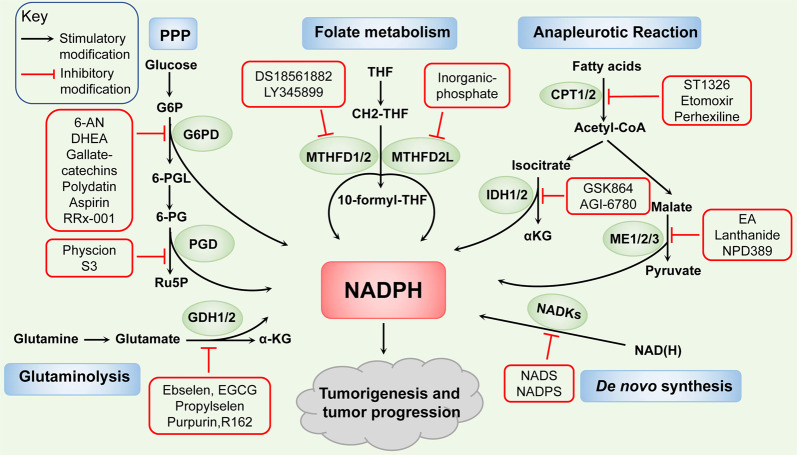
Therapeutic implications for targeting NADPH metabolism. Many inhibitors targeting NADPH-synthesis enzymes have been discovered to impair NADPH pool, thus attenuate tumorigenesis and tumor progression. Such as NADS, NADPS of NADKs. 6-AN, DHEA, gallate-catechins, polydatin, aspirin, RRx-001 of G6PD. Physcion, S3 of PGD. DS18561882, LY345899 of MTHFD1/2. Inorganic phosphate of MTHFD2L. GSK864 of IDH1 and AGI-6780 of IDH2. Lanthanide of ME1 and EA, NPD389 of ME2. ST1326, Etomoxir of CPT1, and perhexiline of CPT2. Ebselen, EGCG, propylselen of GDH1/2 and purpurin, R162 of GDH1

Compared with their normal counterparts, many types of cancer cell have increased oxidative stress and the upregulation of antioxidant capacity. With the metabolic reprogramming of NADPH, cancer cells increase the demand of NADPH for antioxidative effects and anabolic reactions. The specific vulnerability of tumor cells leveraging the aberrant NADPH-synthesis pathways can be exploited to induce cell death under various cellular stresses. Manipulating ROS levels by redox modulation is a way to selectively kill cancer cells without causing significant toxicity to normal cells. This strategy is the basis for many anticancer therapeutics, including chemotherapeutics, radiotherapies, and most small-molecule inhibitor-based therapies, which impair tumor metabolism and induce excessive ROS accumulation, inducing cell toxicity and death.^11,174^ As illustrated in Fig. 3, the inhibitors targeting NADPH-synthesis enzymes are being extensively developed. The specific target, anti-tumor effect, and clinical progress of these inhibitors targeting NADPH metabolism are also summarized in Tables 1 and 2.

**Table 1:** The pre-clinical studies with inhibitors targeting NADPH metabolism in cancer

Target	Compound	Cancer	Concentration range	IC_50_ for tumor cell	IC_50_ for normal cell	Refs.
NADK	Thionicotinamide	Colorectal cancer	10–100 μM	~75 μM (c85, 96 h)	LD_50_ > 800 mg/kg	^38^
G6PD	DHEA	Hepatocellular carcinoma	5–100 μM	~75 μM (HEP-G2, 72 h)	Not available	^183^
	6-AN	Bladder cancer	5–10 μM	~16 μM (TCCSUP, 24 h)	Not available	^179^
	Polydatin	Breast cancer	10–70 μM	17 µM (MCF7, 48 h)	LD50 > 200 mg/kg	^181^
	Aspirin	Breast cancer	0.25–2.5 mM	0.69 mM (SKBR3, 48 h)	0.5 mM (WI 38,48 h)	^182^; Bioorg. Med. Chem. Lett. 22, 3168–3171 (2012)
	RRx-001	Colorectal cancer	5–100 μM	~50 μM (CaCo_2_, 72 h)	Not available	^183^
6PGD	Physcion	Leukemia	10–40 μM	~40 μM (K562, 48 h)	>100 μM (HDF, PIG1, 48 h)	Nat. Cell Biol. 17, 1484–1496 (2015)
MTHFDs	LY345899	Colorectal cancer	10 μM	~12 μM (SW620, 72 h)	>200 μM (CCD-112, 72 h)	^74^
MTHFD2	DS18561882	Breast cancer	140 nM	140 nM (MDA-MB-231, 24 h)	Not available	^186^
GDHs	Propylselen	Lung cancer cells	0–10 μM	3.4 μM (A549, 48 h)	Not available	^187^
	R162	Non-small cell lung carcinoma	20–40 μM	10 μM (KG1a, 48 h)	>40 μM (MRC-5, HFF, 48 h)	^151^
IDH1	GSK864	Glioma	5–100 μM	5 μM (LN382, 48 h)	Not available	^125^
IDH2	AGI-6780	Non-small cell lung carcinoma	0–50 μM	~5 μM (A549, 24 h)	>50 μM (MRC-5, 48 h)	^134^
ME1	Piperazine-1-pyrrolidine-2,5-dione	Colorectal cancer	50 μM	<50 μM (HCT116, 24 h)	>50 μM (IEC6, 24 h)	^90^
ME2	Embonic acid	Non-small cell lung carcinoma	1–10 μM	1.4 μM (H1299, 48 h)	Not available	^189^
CPTs	Perhexiline	Colorectal cancer	10–40 μM	<20 μM (HCT116, 24 h)	>20 μM (CCD841, 24 h)	^167^
CPT1	Etomoxir	Prostate cancer	50–200 μM	<75 μM (VCaP, 48 h)	>75 μM (BPH-1, 48 h)	Mol. Cancer Ther. 13, 2361–2371 (2014)
CPT1A	ST1326	Burkitt’s lymphoma	1–50 μM	8.6 μM (Raji, 72 h)	47 μM (GM130C, 72 h)	J. Natl Cancer Inst. 105, 489–498 (2013)

IC_50_: Half-maximal inhibitory concentration, LD_50_: median lethal dose

**Table 2:** The clinical trials with inhibitors targeting NADPH metabolism in cancer

Target	Inhibitor	Tumor type	Phase	Clinical trial ID	Recruitment status
G6PD	RRx-001	Malignant solid tumor lymphoma	Phase 1	NCT02518958	Completed
	RRx-001	Lymphomas	Phase 1	NCT01359982	Completed
	RRx-001	Small cell cancer	Phase 3	NCT03699956	Active, not recruiting
	RRx-001	Colorectal neoplasms	Phase 2	NCT02096354	Active, not recruiting
G6PD	DHEA	Breast cancer	Phase 3	NCT01376349	Completed
	DHEA	Breast cancer	Phase 3	NCT01376349	Completed
	DHEA	Multiple myeloma and plasma cell neoplasm	Phase 3	NCT00006219	Completed
G6PD, 6PGD, IDH, GDH	EGCG	Colon cancer	Early Phase 1	NCT02891538	Recruiting
	EGCG	Breast neoplasms	Phase 2	NCT02580279	Enrolling by invitation
	EGCG	Lung neoplasms	Phase 2	NCT02577393	Enrolling by invitation
GDH	Ebselen	Hearing loss/cancer	Phase 1	NCT01452607	Completed
	Ebselen	Lung cancer head and neck cancer	Phase 2	NCT01451853	Unknown
IDHs	AG-881	Glioma with an IDH1 or IDH2 mutation	Phase 3	NCT04164901	Recruiting
	BAY1436032	Leukemia, myeloid, acute with IDH1 mutations	Phase 1	NCT03127735	Completed
IDH1	AG-120 (Tibsovo)	Advanced hematologic malignancies with an IDH1 mutation	Phase 1	NCT02074839	Approved
	IDH305	Advanced malignancies with IDH1 mutations	Phase 1	NCT02381886	Active, not recruiting
	FT-2102	Tumors with IDH1 mutations including: glioma chondrosarcoma, hepatobiliary tumors	Phase 1/2	NCT03684811	Active, not recruiting
IDH2	AG-221 (Enasidenib)	Hematologic neoplasms with an IDH2 mutations	Phase 1/2	NCT01915498	Approved

For de novo NADPH synthesis pathway, correlation studies have revealed that thionicotinamide adenine dinucleotide (NADS) and thionicotinamide adenine dinucleotide phosphate (NADPS), converted from the pro-drug thionicotinamide (TN), act as inhibitors of NADKs through targeting the NAD-binding site of NADKs and decreasing the levels of NADPH.^37^ Combining TN with several chemotherapeutic drugs induces synergistic cell killing, indicating its efficacious antitumor effect in DLBCL and colon cancer.^38^ Further, reduced NADPH levels induced by NADPS results in accelerated degradation of DHFR and impairment of the folate cycle, which delays cancer cell growth.^175^

For the PPP enzymes, recent studies have discovered some inhibitors targeting on G6PD, such as NADP^+^ analogs, the competitive inhibitor 6-aminonicotinamide (6-AN), noncompetitive inhibitors epiandrosterone and dehydroepiandrosterone (DHEA) which reduces the availability of NADPH and inhibits the cell growth.^176^ The combination of cisplatin and 6-AN optimizes the clinical dose and minimized the side effects.^177–179^ The new small molecule inhibitors are gradually being discovered, such as, gallated catechins (EGCG, GCG, ECG, CG), as the competitive inhibitors of NADP^+^, repress the activity of G6PD and suppress NADPH production.^180^ The natural molecule polydatin increases the NADP^+^/NADPH ratio and decreases the invasion of breast cancer cells by inhibiting G6PD activity.^181^ Further, the activity of G6PD is also repressed by aspirin casing acetylation of G6PD to decrease the activity of G6PD and the generation of NADPH, and by RRx-001, a novel clinical-stage chemosensitizer and radiosensitizer, which exerts antiproliferative effects in human tumor cells.^182,183^ Moreover, physcion and its derivative S3, novel small-molecule PGD inhibitors which fits in a pocket of PGD near the binding site of 6-PG to inhibit PGD enzyme activity and then decrease the NADPH level, exhibit excellent anticancer effects and sensitize leukemia cells to antimalarial agent dihydroartemisinin (DHA).^184^ For the folate metabolism pathway, the NADP^+^-dependent dehydrogenase activity of MTHFD2 and MTHFD2L can be inhibited by inorganic phosphate.^185^ Besides, other MTHFD2 inhibitors have been reported, including DS18561882 and LY345899 in a substrate-based manner, and treatments based on them decrease cellular NADPH/NADP^+^ ratio, increase cellular ROS levels, and impair tumorigenesis and metastasis.^74,186^ For the glutamine metabolism pathway, ebselen, epigallocatechin-3-Gallate (EGCG), and propylselen are reported to bind to GDH-active sites to abolish NADP^+^ binding and impair in cancer cell functions.^187^ A study also shows that purpurin and its analog, R162, acting as mixed model inhibitors of GDH1, inhibit GDH1 activity, elevate ROS levels and thus attenuate cancer cell proliferation.^151^

For the NADPH-synthesis enzymes involved in anapleurotic reactions, including IDH1/2, ME1/2/3, and CPT1/2, the targeting inhibitors are also being extensively developed. Study shows that treatment with GSK864 as IDH1 inhibitor binding an allosteric site on IDH1 reduces the NADPH/NADP^+^ ratio and prolongs the survival of glioblastoma multiforme (GBM) PDXs model.^125^ AGI-6780 treatment, binding with IDH2 or mutant IDH2 in an allosteric manner at the dimer interface, reduce the IDH2 activity and lead to the repression of cell growth in lung cancer.^134^ Mutant IDH-targeted therapy and a number of important recent pre-clinical and clinical studies in IDH-mutant solid tumors have been extensively reviewed elsewhere,^147^ and listed in Table 2. Furthermore, NPD389 binding to ME2 in fast-binding mode impairs its activity,^188^ and embonic acid (EA) induces the cellular senescence of H1299 cancer cells through its noncompetitive inhibitory activity against ME2.^189^ Further, ME1 treated with the inhibitor (piperazine-1-pyrrolidine-2,5-dione) has little effect on normal rat intestinal epithelial cells but strongly suppresses human CRC cell growth by targeting ME1 NADP^+^-binding site and reducing the NADPH level.^90^ Lanthanide treatment represses cell proliferation and the epithelial–mesenchymal transition (EMT) by inhibiting ME1 in oral squamous cell carcinoma cells.^93^ In addition, CPTs are also considered to be targeted. Glioma cells with FAO inhibited by etomoxir, a CPT1 inhibitor, exhibits a profound decrease in NADPH levels, reduced GSH content and elevation of intracellular ROS levels. Besides, CPT1A-suppression or etomoxir treatment fails to maintain redox homeostasis in detached CRC cells and induces sensitivity to glucose deprivation in PDAC cells.^166,190^ Further, in gastrointestinal cancer cells, genetic inhibition or pharmacological treatment of CPT2 with perhexiline disrupts NADPH and promotes cell apoptosis after oxaliplatin treatment. Combining perhexiline with oxaliplatin leads to a significant suppression of cancer progression.^167^ Other inhibitors of CPTs are also discovered, such as Ro25-0187, ST1326 which are expected to be used for cancer treatment.^191^

## Conclusions

In summary, the essential role of NADPH homeostasis has been increasingly recognized in cancer development and progression through cellular antioxidative effects and anabolic reactions. Pharmacological restriction of cellular NADPH availability by targeting its synthesis pathways to impair NADPH homeostasis is currently recognized as a crucial and potential strategy for cancer treatment.

However, there is an interdependent relationship in which the NADPH pool is simultaneously supported and used by various pathways in cells. For example, pyruvate kinase muscle isoform 2 (PKM2) inactivation can both attenuate the glucose flux to PPP and enhance folate metabolism to mediate NADPH generation.^32,43^ Moreover, because of the heterogeneous nature of tumors, there are considerable variations in NADPH-related processes in different tumors, for example, the main pathways of glutamine metabolism in the context of PDAC are different from the previous prevailing view as informed by studies of other cancers,^159,192^ indicating the need for careful analyses of individual characteristics among cancers for establishing individualized precision therapy. Moreover, the special functions of these metabolic enzymes are not fully understood in cancer. For instance, the reverse-mode NNT that consumes NADPH to support NADH and ATP productions in contrast to the conventional view has not been reported with respect to cancer.^113^ Besides, because of the high plasticity of the metabolic network and metabolite exchange among cancer and stromal cells, a compensatory response can be readily induced to produce limiting metabolites.^193^ In addition, the relative contribution of these pathways and enzymes to NADPH production can be variable in different cell types and under different conditions. Hence, additional studies are needed to evaluate the entire NADPH metabolome, identify the important interrelationships and determine the main pathway to select more suitable targets. Also, the effects of NADPH metabolism on immune cells in the tumor microenvironment are needed to explore for exploiting novel anticancer opportunities.

As the NADPH metabolism are shared in normal and cancer cells, selectively targeting NADPH synthesis under special circumstances without affecting normal cells is difficult. Therefore, one of the greatest challenges to target cancer metabolism is the induction of toxic effects on noncancerous cells. Further, many reported small-molecule inhibitors target several metabolic enzymes with similar structures, for example, EGCG targets both NADPH-dependent FASN and NADP^+^-dependent GDH.^21,187^ The functions can be also markedly different among the isoforms of these enzymes. For instance, cytosolic ALDH1L1 mainly regulates reduced synthesis, while mitochondrial ALDH1L2 produces NADPH to attenuate oxidative stress.^78^ IDH1/2 use NADP^+^ as a cofactor while IDH3 needs NAD^+^^121^. The development of highly selective or isoform-specific inhibitors will reduce side effects and is an important goal for the near future. Most compounds specifically targeting cancer NADPH metabolism are in preclinical studies, thus there are still challenges to address before these compounds enter the clinic. Collectively, to better understand the therapeutic potential of NADPH metabolism, more preclinical and clinical studies should be implemented to address these difficulties, and combined approaches with immunotherapy and/or chemotherapeutics should be pursued as the best strategies because of their synergistic effects.
